# Uncertainty quantification for probabilistic machine learning in earth observation using conformal prediction

**DOI:** 10.1038/s41598-024-65954-w

**Published:** 2024-07-13

**Authors:** Geethen Singh, Glenn Moncrieff, Zander Venter, Kerry Cawse-Nicholson, Jasper Slingsby, Tamara B. Robinson

**Affiliations:** 1 Department of Botany and Zoology, Centre for Invasion Biology, Stellenbosch University, Stellenbosch, South Africa; 2 Global Science, The Nature Conservancy, Cape Town, 7945 South Africa; 3 Department of Statistical Sciences, Centre for Statistics in Ecology, Environment and Conservation, University of Cape Town, Private Bag X3, Rondebosch, Cape Town, 7701 South Africa; 4 Norwegian Institute for Nature Research—NINA, Sognsveien 68, 0855 Oslo, Norway; 5 Carbon Cycles and Ecosystems, Jet Propulsion Laboratory, California Institute of Technology, Pasadena, CA USA; 6 Department of Biological Sciences and Centre for Statistics in Ecology, Environment and Conservation, University of Cape Town, Private Bag X3, Rondebosch, Cape Town, 7701 South Africa; 7 Fynbos Node, South African Environmental Observation Network, Centre for Biodiversity Conservation, Cape Town, South Africa

**Keywords:** Satellite, Remote sensing, Machine learning, Conformal prediction, Uncertainty quantification, Environmental sciences, Forestry, Computer science, Software

## Abstract

Machine learning is increasingly applied to Earth Observation (EO) data to obtain datasets that contribute towards international accords. However, these datasets contain inherent uncertainty that needs to be quantified reliably to avoid negative consequences. In response to the increased need to report uncertainty, we bring attention to the promise of conformal prediction within the domain of EO. Unlike previous uncertainty quantification methods, conformal prediction offers statistically valid prediction regions while concurrently supporting any machine learning model and data distribution. To support the need for conformal prediction, we reviewed EO datasets and found that only 22.5% of the datasets incorporated a degree of uncertainty information, with unreliable methods prevalent. Current open implementations require moving large amounts of EO data to the algorithms. We introduced Google Earth Engine native modules that bring conformal prediction to the data and compute, facilitating the integration of uncertainty quantification into existing traditional and deep learning modelling workflows. To demonstrate the versatility and scalability of these tools we apply them to valued EO applications spanning local to global extents, regression, and classification tasks. Subsequently, we discuss the opportunities arising from the use of conformal prediction in EO. We anticipate that accessible and easy-to-use tools, such as those provided here, will drive wider adoption of rigorous uncertainty quantification in EO, thereby enhancing the reliability of downstream uses such as operational monitoring and decision-making.

## Introduction

Global accords such as the United Nations (UN) Sustainable Development Goals (SDGs) and Convention on Biological Diversity (CBD) rely on data to set targets, monitor progress, and allocate limited resources. Some of these datasets are obtained by applying machine learning and Artificial Intelligence (AI) methodologies to geospatial data (GeoAI), particularly Earth Observation (EO) data^1,2^. For example, the Group on Earth Observations has identified which SDGs are quantifiable, to some extent, through EO data^1,3^. Additionally, efforts have been undertaken to define more directly measurable variables that enhance biodiversity indicators^4^, exemplified by the Essential Biodiversity Variables (EBV)^2^. Despite the benefits of these datasets, they inherently contain uncertainty. Therefore, data-driven decision-making processes should consider this uncertainty. If the uncertainty of the data underpinning a decision is too high, its utility for decision-makers diminishes. Similarly, if the uncertainty is not correctly quantified and presented, it may result in suboptimal decision outcomes.

### Geospatial artificial intelligence (GeoAI) in ecology and environmental management

Historically, ecological observations have been made by experts in the field, over limited spatial extents with few or no revisits. The emerging field of GeoAI holds great promise for revolutionizing conservation and environmental management^5^. It has the potential to enhance the capabilities of ecologists by increasing their field of view and frequency of observation at a lower cost than equivalent field work^5^. However, there exists a gap between the research priorities of scientists and the practical requirements for informed decision-making^6^. Among the contributing factors are end-user trust and the point-prediction nature of EO-derived datasets that have limited flexibility to meet the needs of end-users. For instance, there are regions of the world that are critically under-sampled, which may not be well characterized by models trained on data from the global North, unknowingly limiting the suitability of the derived datasets for end-users and compromising their trust^7^.

In these scenarios, Uncertainty Quantification (UQ) stands to benefit both data creators and data users. For data creators, discerning and flagging unreliable predictions can lead to a better understanding of a model’s biases and errors leading to targeted data collection, the development of improved models and GeoAI systems with reduced uncertainty, wider adoption and improved efficiency. Some UQ methodologies may increase downstream flexibility by advocating for continuous probabilistic predictions. For instance, end-users can select a threshold probability better aligned with their spatio-temporal context and the associated real-world costs of omission and commission errors. For data users, the communication of prediction uncertainty could enhance trust^8,9^, and help mitigate the negative consequences associated with the over-reliance on unreliable predictions that could lead to erroneous decision making^10^. Despite the benefits to be gained from UQ, a widely adopted framework that is reliable, flexible and easy-to-use is absent in the field of machine learning, let alone in EO^8,11^.

### Uncertainty and its quantification in earth observation (EO)

Data acquired by satellites, including reflectance spectra, backscatter or waveform data, contain inherent uncertainty owing to measurement noise, randomness^12^, sensor anomalies (for example, Landsat-8 thermal calibration issues^13^), inaccurate pre-processing steps (for example, atmospheric correction, orthorectification and terrain corrections) and partial data acquisition (for example, due to the scan-line error in Landsat-7 or the acquisition footprint of GEDI^14^). These sources of uncertainty represent irreducible error and are denoted as aleatoric uncertainty^15^. In addition, uncertainty that arises from the lack of knowledge or understanding of a system, the selected modelling framework and through the stochastic nature of model fitting are cumulatively referred to as epistemic uncertainty^15^. Both categories of uncertainty are considered in this study.

Uncertainty is distinct from error/accuracy, uncertainty quantification defines the estimated distribution within which the true value lies and corresponds to the confidence in a prediction. Conversely, error is defined as the difference between an observed true value and a model prediction. The disclosure of uncertainty information alongside prediction error constitutes a complementary practice^16^. This synergy is underpinned by the inherent sparsity of error data, as error is quantified solely in the presence of reference data. In contrast, uncertainty information can be systematically reported for all pixels, regardless of the sparse availability of reference data.

Given the benefits of UQ and its long-perceived lack in EO, we aimed to provide empirical evidence through a systematic literature review of all national to global datasets catalogued in the Google Earth Engine (GEE) and GEE community catalogues^17^. Next, we make the case for rigorous uncertainty quantification through conformal prediction and go on to extend the availability of conformal prediction to GEE through the introduction of widely applicable and easy-to-use modules. Third, to demonstrate the versatility and computational efficiency of the introduced JavaScript and Python tools, we apply the introduced modules to three high-valued EO applications, specifically, land cover classification, tree canopy height estimation and the detection of invasive tree species that encompass traditional machine learning and deep learning workflows, and classification and regression tasks that cover local to global extents. Lastly, we discuss the opportunities offered by conformal prediction techniques that are likely to advance EO and future operational monitoring systems.

## Literature review

### Assessing the status of uncertainty quantification (UQ) in earth observation (EO)

To assess the current status of different UQ methods in EO, we examined all machine learning derived datasets in the GEE and the GEE community catalogue with the latest update on the 2 November 2023 considered^17^ (Table S1-3). These data catalogues were selected since they contain commonly used datasets with a national to global coverage. For the core GEE catalogue, “machine learning”, “uncertainty”, “UQ”, “prediction interval”, “credible interval”, “confidence interval”, “variance” and “standard deviation”keywords were used to find and filter all machine learning derived datasets that were reviewed. If a dataset was not tagged with one of the keywords or quantified uncertainty but was derived using expert rule-based systems or statistical models, they were not considered. In addition to examining the band information, the full-text research paper or associated theoretical documentation was examined to determine if i) machine learning was used to derive the dataset, ii) uncertainty was quantified for the derived dataset and, if it was quantified, iii) which method was employed. Overall, 243 datasets were assessed.

### The status of UQ in earth observation (EO)

**Figure 1:**
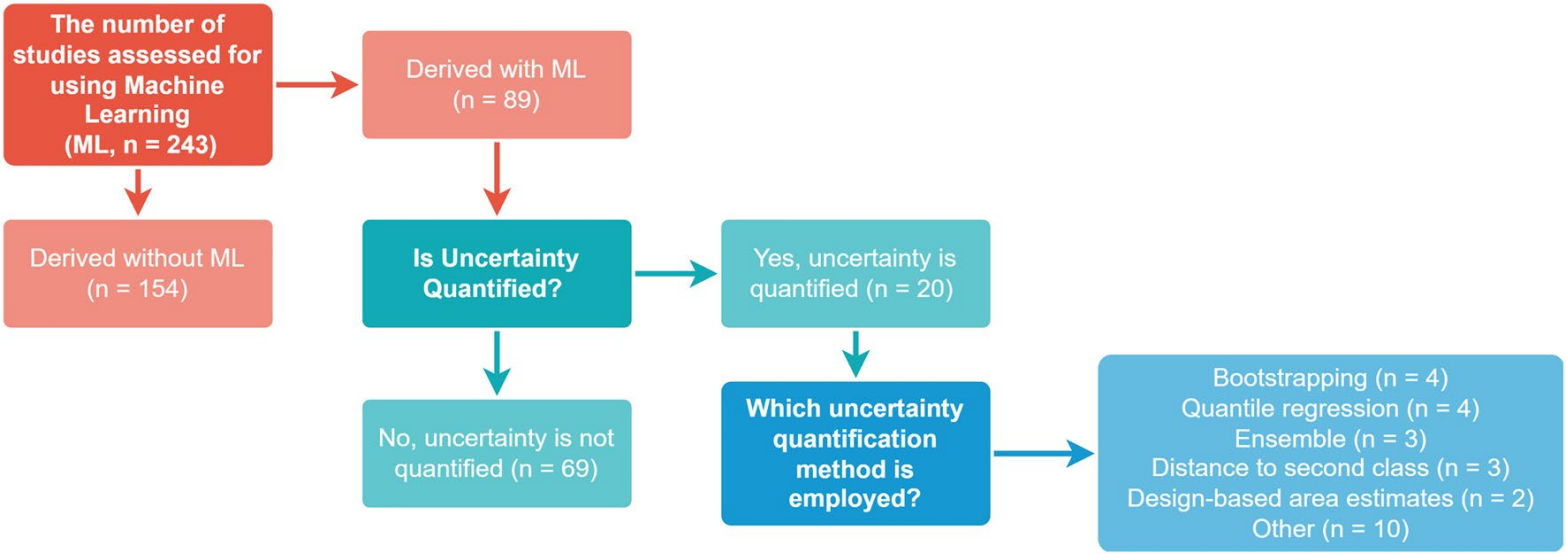
The classification of each assessed dataset (n = 243) from both the Google Earth Engine (GEE) catalogue and the GEE community catalogue. Only the datasets that used machine learning were considered from the main GEE catalogue. For the community catalogue, all datasets comprising the catalogue up to the 2 November 2023 update were considered. Five studies used more than one UQ method.

Uncertainty is seldomly considered in EO with only 20 of the 89 (22.5%) reviewed datasets in the GEE catalogues citing studies that quantified uncertainty (Fig. 1). Some research efforts conflated the concepts of error and uncertainty (for example,^18,19^) and were not considered further. In addition, an evaluation of the quality (validity and efficiency) of the quantified uncertainty was notably lacking, except for its partial consideration in two studies (i.e., SoilGrids v2.0. and tree canopy height estimation^20^). These observations emphasize the need for increased understanding about UQ in EO and underscores the importance of UQ frameworks that are accessible, easy-to-use and support a wide range of machine learning tasks.

For the 20 studies that performed UQ, model ensembles that capture variance in probability-like scores are commonly used for classification tasks while quantile regression is commonly used for regression tasks. While conventional ensemble methods that rely on assessing the prediction variance among multiple model predictions are widely employed in predictive modelling for pixel-wise UQ, they only capture epistemic uncertainty^11^. Moreover, their operational deployment is associated with high computational costs given the need to train multiple models. Analogous to ensemble methods employed in classification and regression tasks, the derivation of pixel-wise uncertainty in regression tasks can be achieved through quantile regression. However, it does not always expressly guarantee the coverage stipulated by the quantile levels. For example, A quantile regression model incorporating predictions at the lower 5th and upper 95th quantiles often prove inadequate in encompassing the true value within 90% of the prediction intervals^21^. Bootstrapping and design-based area estimates are commonly used to provide confidence intervals around accuracy scores and area coverage, respectively. Nevertheless, both methodologies, by design, fall short by not providing pixel-wise prediction intervals and are not yet readily accessible owing to the specialised knowledge requirement and lack of tooling and software. Notably, many datasets that employ UQ relate to the estimation of forest structure and carbon sequestration.

### Conformal prediction: a potential solution

Conformal prediction is a mathematical framework that can quantify both epistemic and aleatoric uncertainty in combination^22^. It is amenable to integration with any prediction model and dataset, irrespective of its statistical distribution, while adhering to a pre-specified confidence level^22–24^. This translates to the provision of uncertainty estimates with a constrained error rate or tolerance level. For example, if a 95% confidence level, corresponding to a 5% tolerance level, is specified, 95% of the prediction regions provided by a conformal predictor will contain the true value (for classification problems, this prediction region corresponds to a set of labels, a multilabel prediction)^22,23^. This coverage guarantee is referred to as the validity property of conformal predictors and remains conspicuously absent from all other pixel-wise UQ methodologies, except under certain strict distribution assumptions^24,25^. The fulfilment of the validity property of conformal prediction hinges on the exchangeability assumption being satisfied. Exchangeability signifies that the data utilized for calibrating the conformal predictor could be swapped with the data during inference without affecting the probability distribution of the target variable to be estimated.

Fulfilling the validity property in isolation proves inadequate because a broad prediction region or a set encompassing all candidate classes will meet the coverage criteria but will be uninformative. Hence, the statistical efficiency pertaining to the length or width of the prediction regions in classification and regression tasks becomes important, necessitating smaller prediction set sizes and narrow prediction intervals. In instances where a prediction fails to reach the stipulated confidence level, a null prediction may be provided. For instance, this may be the case for remnant cloud pixels produced by imperfect cloud masking. Alternatively, when the prediction region is too large to be informative, the corresponding prediction can be flagged for human intervention^22^. In this way, conformal prediction can serve as a quality control system devoted to dependable predictions.

There are three primary types of conformal prediction; split or inductive conformal prediction, jackknife+, and transductive conformal prediction^23^. This study focuses on split conformal prediction due to its computational efficiency and subsequent suitability for large-scale EO applications. In contrast, jackknife+ and transductive conformal prediction methods are more computationally intensive as they utilize cross-validation strategies^26^, with transductive conformal prediction being equivalent to performing conformal prediction with a leave-one-out cross-validation strategy. Although these methods are more computationally demanding, they are more sample-efficient and offer stronger coverage guarantees compared to split conformal prediction, making them suited to limited data settings.

### Conformal prediction: the six steps

Practically, producing uncertainty estimates using conformal prediction involves six steps: the initial procedural phase entails the partitioning of a given reference dataset into three subsets, namely the training set, the calibration set, and the test set^22,23^. Subsequently, a predictive model is trained on the training set, after which it is deployed to estimate the class probabilities or regressed values within the calibration and test sets. In the third step, during the calibration phase, each calibration instance is scored based on its nonconformity with the true value. An example of a simple but common scoring function for classification tasks is hinge loss and encompasses the subtraction of one from the classifier-produced probability-like scores^23^. Next, as part of the calibration stage, these nonconformity scores are used to compute a probability threshold corresponding to the user-defined confidence level (1-alpha) after a finite-sample correction (Eq. 1,^23,27^).

1 $$qLevel=\frac{ceil \left(\left(1+nCal\right)*\left(1-\alpha \right)\right)}{nCal} ,$$

where *qLevel* corresponds to the adjusted quantile level, *alpha (α)* corresponds to the proportion of acceptable errors or tolerance level and *nCal* denotes the size of the calibration set. In the fifth step, post-calibration and during the inference stage, the computed probability threshold value is used in the creation of class sets for each test instance. All classes with nonconformity scores that are greater than or equal to the probability threshold value are included in the output prediction set^28^. This corresponds to the inclusion of class labels in the prediction set if their associated confidence exceeds a desired and user-specified confidence level^22^.

In the final step, the test set is used in the evaluation stage to assess the validity and efficiency of the calibrated conformal predictor by computing the empirical marginal coverage and the average set size, respectively. For instance, if the specified confidence level is 95%, then ~ 95% of the prediction sets for the out-of-sample test instances, across all classes, should include the true class. If the coverage deviates from the specified confidence level, this implies a violation of the exchangeability assumption through, for example, quantifying uncertainty in a region that was not represented in the training and calibration data. This assumption assumes the nonconformity scores between the calibration and test sets are permutation invariant and thus, their ranks being uniformly distributed^23,28^.

For regression tasks, the six steps remain unchanged with the exception of the scoring function used to generate the nonconformity scores and the method used to evaluate the prediction regions. The most used scoring function in regression computes the absolute residual for each calibration and test instance. During the inference stage, the absolute residual corresponding to the user-specified confidence level is added and subtracted from the mean prediction values to provide an upper and lower bound, respectively. A drawback of this simple scoring function is the lack of adaptability i.e., all prediction intervals have the same width. Therefore, conformal quantile regression has been introduced to provide adaptability whereby more difficult prediction instances have wider intervals than easier prediction instances^21,23^.

### Demonstrating the utility of conformal predictors

**Figure 2:**
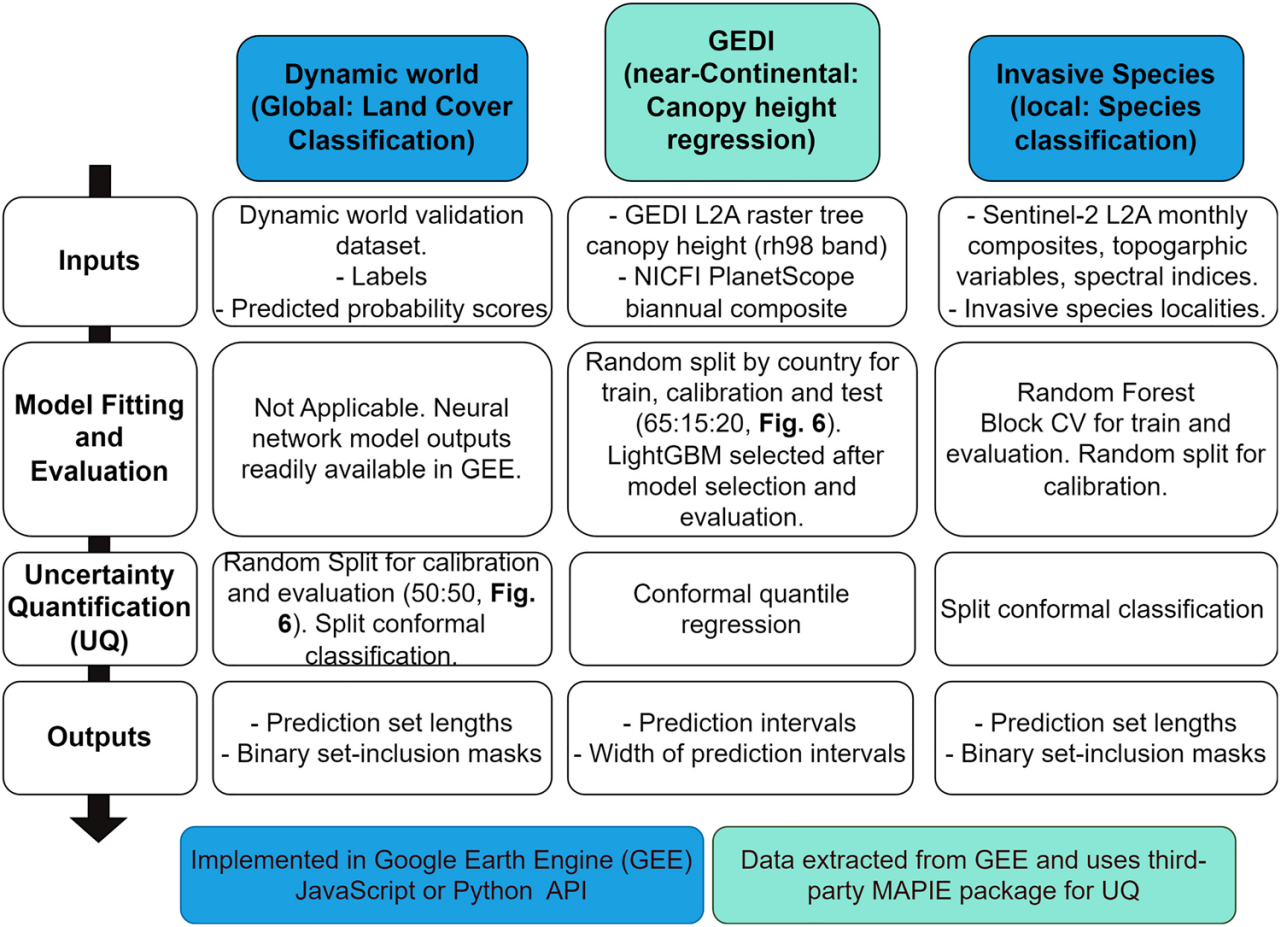
Summarising the workflow for each of the three case studies including uncertainty quantification using the Google Earth Engine (GEE) JavaScript code editor API (blue) and the GEE Python API with the Python MAPIE package (turquoise).

Conformal prediction has been attracting growing attention; however, it has yet to be widely adopted within the domain of GeoAI^11,29^. A few recent studies have ventured into the application of conformal prediction to EO data, with a principal focus on verifying its validity and efficiency property in the context of spatial autocorrelation for classification tasks across small extents^11,29^. Through the case studies described below, we extend previous work by simultaneously demonstrating the versatility, scalability, and validity of conformal prediction. Each application can be characterised by a distinct set of properties that range from relying on small to large dataset sizes encompassing local to global extents and both regression and classification tasks that use GEE image or feature collections.The case studies look at quantifying uncertainty for invasive tree species mapping for a region in South Africa, canopy height estimation based on Global Ecosystem Dynamics Investigation (GEDI) for Africa^30^, and land cover classification using the global Google Dynamic World dataset^31^. We consider applications with small (< 1500 instances) to large datasets (> 110 M instances), for both classification and regression and both GEE image and feature collections, which are used in deep-learning models and traditional machine learning models, respectively. The steps taken to produce the uncertainty information in each case study is summarised below (Fig. 2). For both classification tasks, we use the least ambiguous set-valued conformal classifier method^27^. For the canopy height regression case study, we use conformal quantile regression^21^. Although the tolerance level can be set to a low value, setting it to the minimum (zero) results in large and uninformative prediction regions. Consequently, we select a tolerance of 10% for both classification tasks and 5% for the regression task.

### Global google dynamic world (classification)

**Figure 3:**
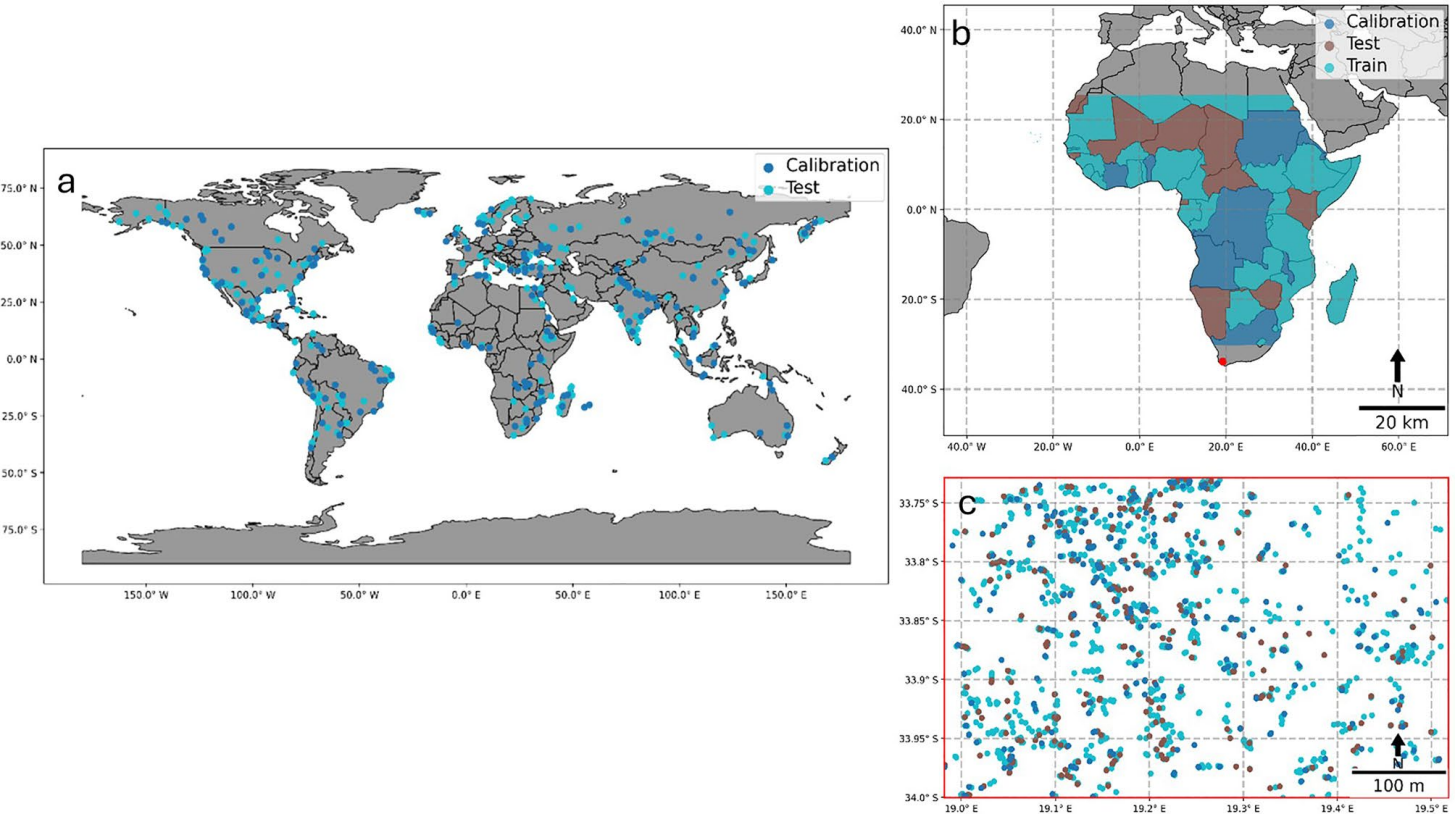
The distribution of train (if applicable), test and calibration samples for (**a**) Land cover classification using Dynamic World, randomly split (80:20) calibration and test samples derived from the 409 out-of-sample validation samples made available by Google. Each sample represents an image with 512 × 512 pixels. (**b**) Canopy height estimation using GEDI randomly split by country for the train (> 50 M points), test (> 31 M points) and calibration (> 32 M points) samples (65:20:15). The countries considered have been limited to the extent of the NICFI Africa PlanetScope dataset and (**c**) Invasive tree species classification randomly split (65:20:15). The map was generated with the geemap v0.29.4. (https://geemap.org/,^32^), and matplotlib v3.8. (https://matplotlib.org/,^33^) software packages.

Google’s Dynamic World dataset is a near-real time land cover product produced for every Sentinel-2 scene with less than or equal to 35% cloud cover. As part of the neural network based landcover model output, a per-class probability band is produced in addition to a discrete class output band corresponding to the class with the maximum probability. The discrete labels for 2020 were assessed to have an overall agreement of 73.8% against expert-derived reference labels^31^. To quantify uncertainty, the globally distributed dynamic world validation set that was released comprised of 409 reference label images (512 × 512 pixels) and corresponding predicted per-class probability bands (refer to^31^, for details on the sampling design) were randomly split into 80% calibration and 20% test data (Fig. 3a). The calibration set was used to calibrate the least ambiguous set-valued conformal classifier, while the test set was used to evaluate the empirical marginal coverage and average set size. Assuming the calibration set scores are exchangeable with the scores derived from any output dynamic world scene, the calibrated conformal classifier can be applied to produce prediction sets that will include the actual class with a high (0.9) probability (empirical marginal coverage = 0.89). Probability threshold values are computed for various confidence levels (0.7 ≤ (1 − *α*) ≤ 0.95, in 5% intervals, Supplementary Table S4).

**Figure 4:**
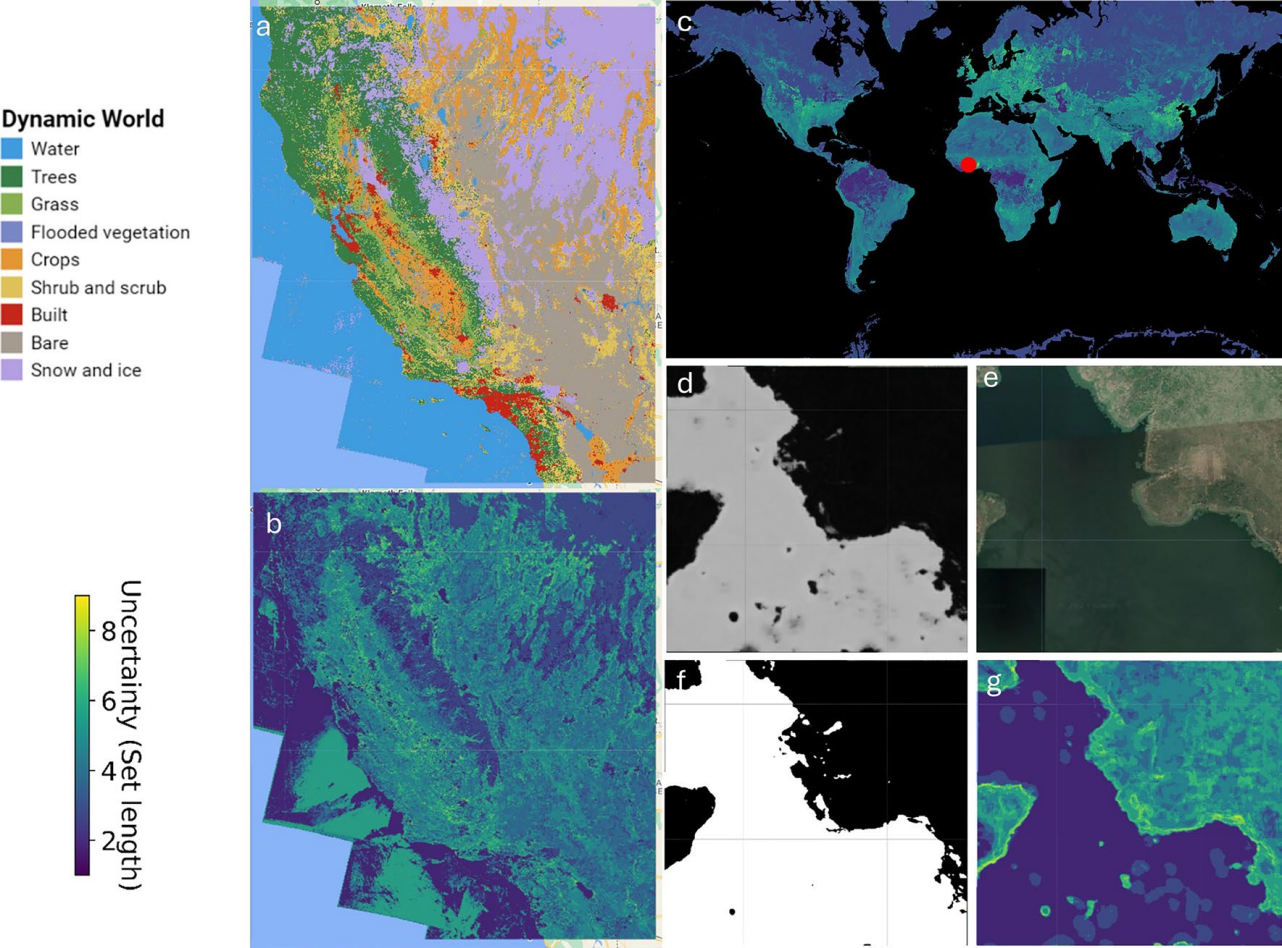
The (**a**) & (**b**) Dynamic World land cover classification with associated uncertainty over California, United States of America showing confident inland water, built-up, snow and ice predictions. (**c**) The global distribution of uncertainty for the first non-null land cover image in 2020. (**d**–**g**) A high-resolution Google Earth Image (red point in C) with corresponding (**e**) probability of water, (**f**) the prediction sets that include the water class and (**g**) the length of the prediction sets. Empty set predictions (length = 0) are not shown. Interactively explore the Dynamic World dataset with accompanying uncertainty information or quantify uncertainty for any Dynamic World scene https://eegeethensingh.projects.earthengine.app/view/conformaluq. The map was generated with the geemap v0.29.4. (https://geemap.org/,^32^), matplotlib v3.8. (https://matplotlib.org/,^33^) software packages and RGB satellite basemap from Google, layers available in Google Earth Engine.

For classification tasks, we represent pixel-wise uncertainty as the number of classes included in a pixels’ prediction set. Highly uncertain predictions can either be represented with an empty set (length equal to zero) or a large multi-label set (length closer to the total number of candidate classes; for instance, nine for Dynamic World). A multi-label set suggests that the prediction model is finding it challenging to distinguish between several possible class labels at the desired confidence level (average prediction set size = 2.5 classes). Higher desired confidence in the prediction leads to larger prediction sets or intervals (analogously to how higher desired confidence in parameter estimates lead to larger confidence intervals). Although such a prediction is not incorrect per se, it is inconclusive, and human intervention would be required to derive the true label. Empty set predictions are examples where the model could not assign any label, typically meaning that the example was very different from the data the model was trained on. Conversely, the most confident predictions are shown with set lengths of one. For instance, inland water and built-up predictions are among the most reliably mapped land cover classes (Fig. 4a, b), whereas object boundaries typical of mixed-landcover pixels, transition and seasonal areas are associated with higher prediction uncertainty and larger prediction set lengths (Fig. 4d–g).

### Continental GEDI canopy height (regression)

NASA’s Global Ecosystem Dynamics Investigation (GEDI) is a space-based laser altimeter with a full-waveform detector that captures the vertical structure and distribution of vegetation, a proxy for biomass and tree canopy height^8^. These volumetric tree-stand variables are captured in 100 relative height (rh) bands. For example, the selected rh98 response band corresponds to the height at which 98% of energy is returned to the detector from a 25 × 25 m footprint area^30^. In this case study, the canopy height product made available in GEE was combined with the former 2020 biannual Visible and Near-Infrared (VNIR) predictors from the NICFI PlanetScope data^34^. Predictors were extracted from GEE for each GEDI instance overlapping the Africa PlanetScope extent. Next, each country in the extent was randomly assigned to a train, calibration, or test set (Fig. 3b). The train set was used to fit a decision tree based Light Gradient Boosting Machine (LightGBM) quantile regression model, thereafter, the calibration set was used to calibrate the quantile regressor. This enabled the provision of canopy height point estimates, together with a prediction interval that contains the actual canopy height value, as measured by GEDI, with a high probability (95%).

**Figure 5:**
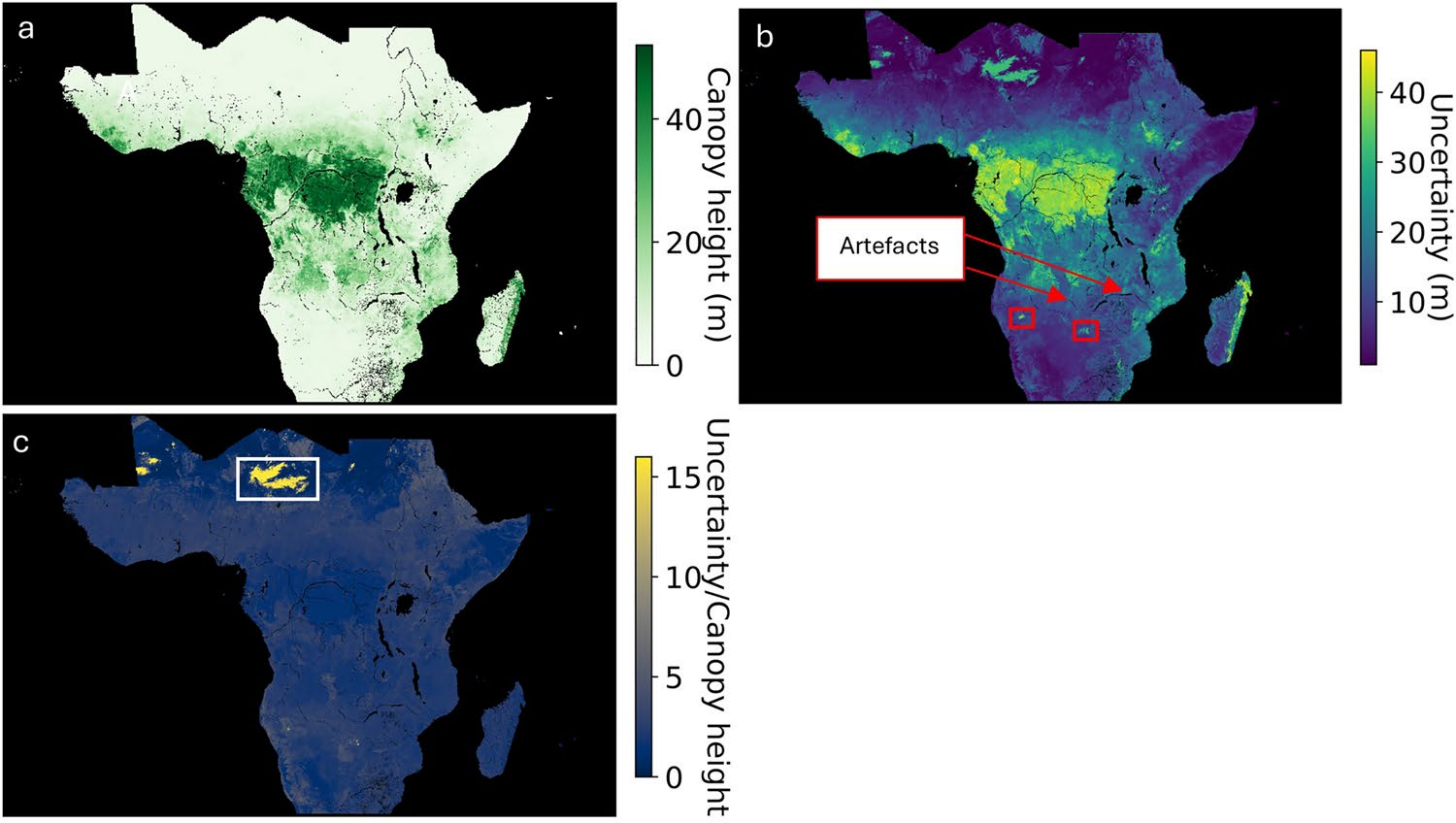
The distribution of (**a**) tree canopy height as estimated from GEDI and NICFI PlanetScope data, (**b**) associated prediction intervals with a 95% confidence level, highlighting diagonal linear artefacts and large prediction intervals for pans (red boxes) and (**c**) the ratio between the two (**a** & **b**, uncertainty/Canopy height), highlighting an area in the Terene desert with high uncertainty and low canopy height. The data can be interactively explored https://ee-geethensingh.projects.earthengine.app/view/conformaluq. The map was generated with the geemap v0.29.4. (https://geemap.org/,^32^), and matplotlib v3.8. (https://matplotlib.org/,^33^) software packages.

For the canopy height regression task (test set RMSE = 3.30 m), we represent uncertainty as the difference between the upper and lower prediction bound, referred to as prediction interval. The prediction interval contains the actual canopy height, as based on GEDI, with a 95% probability (empirical marginal coverage, 95.15% ± 0.07). Prediction intervals with a greater width are representative of high prediction uncertainty and, in this case, may be indicative of the difficulty in EO-based estimation of tall tree canopy as opposed to being attributed to the deficiencies of conformal prediction. The average prediction interval width is 9.28 m ± 0.03 m. Higher canopy height (Fig. 5a) corresponds to wider prediction intervals and greater uncertainty (Fig. 5b), but when one looks at water systems and pans there are instances that deviate from this generalisation (yellow regions in Fig. 5c). There is a marked increase in uncertainties in the context of water systems, pans such as the Sua salt pan in Botswana and the Namibian Etosha pan (Fig. 5b, red boxes), and the Terene Desert shared between Niger and Chad (Fig. 5c, white box). Moreover, diagonal image artefacts (Fig. 5b, feint lines), comprising aleatoric uncertainty due to seamlines, in central Africa also show a similar deviation.

### Local Invasive tree species discrimination (classification)

This case study presents a novel analysis and diverges from the previous Dynamic World case study based on data composition, the extent of interest, and the modelling workflow. Here, the dataset consists of georeferenced locations of species within a GEE feature collection instead of dense image labels like Dynamic World. We also demonstrate an entire workflow that involves model fitting, unlike Dynamic World which only involves UQ owing to the readily available class-wise probability outputs on GEE.

A combination of high-resolution Google Earth Imagery and familiarity with the distribution of the dominant invasive alien tree species in the local area of interest (Fig. 3c, The Western Cape, South Africa)^35^, was used to sample the invasive tree localities and its surrounding landcover. The invasive tree species include Acacia, Eucalyptus and Pinus species, commonly known as wattles, gums and pines respectively. The region is located in the water scarce Western Cape province of South Africa that receives ~ 380 mm rainfall per year^36^. One of the major threats to water security in the upper Berg and Breede catchments is invasive alien trees^35,36^. This case study represents a common scenario whereby managers are interested in invasive tree species within their jurisdiction of influence/mandate. Moreover, the limited size of the dataset (< 1500 instances) and small extent of interest corresponds to a common set of conditions under which satellite remote sensing is used. Similar pilot studies are an important preliminary to scaling up the application of satellite-based mapping and an important milestone to develop reliable large-scale operational monitoring programmes.

The mapping workflow used in this case study (Fig. 2), included, feature preparation, model evaluation and uncertainty quantification. During feature preparation, Sentinel-2 level 2A median monthly composites were created after performing cloud and cloud-shadow masking based on s2cloudless^37^. Thereafter, the masked cloud pixels were imputed using the median values from the temporally uncontaminated-pixel neighbours of a 2-month and 6-month median image. Lastly, we stacked additional spectral indices and topographic covariates that included, elevation, slope, aspect, topographic position index, continuous heat insolation index and derived location variables. Here, the derived location variable refers to a pixel’s coordinates along an axis after being tilted by different angles relative to the X-axis. This supplies radial coordinates to the model that reduces the effects of spatial autocorrelation whilst accounting for spatial patterns^38^. Next, during the model training and hyperparameter tuning phase, a random forest model was trained using a ten-fold spatial cross validation approach. Here, the coordinates of the input tree species localities were clustered and split into folds to limit the effects of spatial autocorrelation. A summed confusion matrix and the cross-validation accuracy statistics are returned. Finally, for the calibration of the conformal predictor and the quantification of uncertainty, we used the least ambiguous set-valued conformal classifier, discussed above, with a model trained on the entire train set. The calibrated classifier can then be used to obtain pixel-wise sets and the corresponding length of the prediction sets with a 90% confidence level.

**Figure 6:**
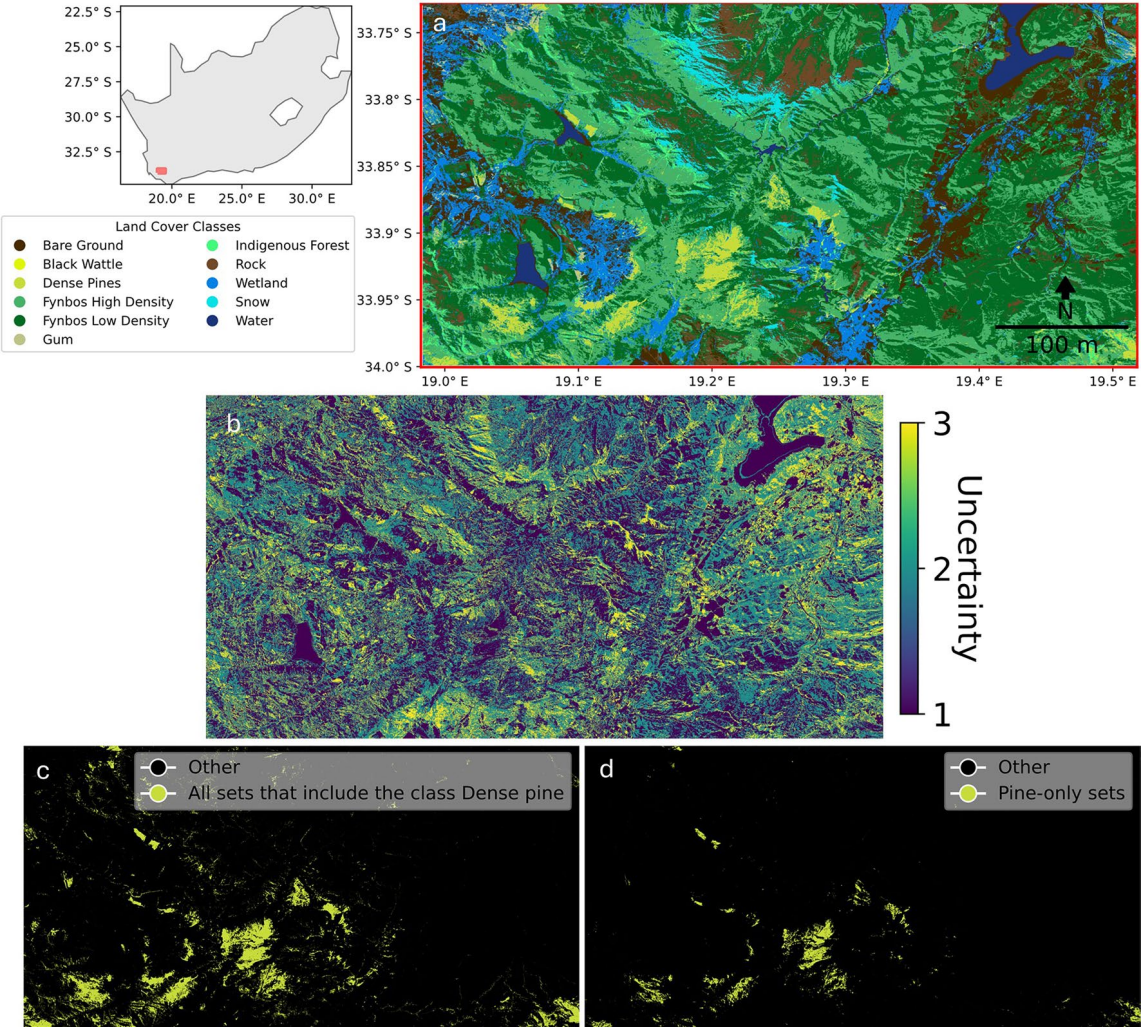
The (**a**) discrete classification of invasive tree species and its surrounding land cover, (**b**) associated uncertainty as presented by the pixels’ prediction set length, (**c**) all pixels that include pine species along with other classes within their predictions sets (set length > 1), and (**d**) pixels that only include the Dense pine class in their prediction sets (set length = 1) with a 90% confidence level. Training, calibration and test data only covered natural lands and hence agricultural and urban areas are associated with high uncertainty. The map was generated with the geemap v0.29.4. (https://geemap.org/,^32^), and matplotlib v3.8. (https://matplotlib.org/,^33^) software packages.

The invasive tree species classifier has an average set size of 1.53 and an empirical marginal coverage of 0.92. The small set size suggests that the model produces mostly confident predictions and that the time series covariates are highly suited for discriminating and localising pine, wattle, and gum tree species. The uncertainty information (prediction sets) of, for example, pine trees could be used to prioritize the inspection sites that may contain pine (set length > 1) or to find pine infested areas, with a 90% probability, for intervention efforts (set length = 1, Fig. 6).

## Discussion

Uncertainty quantification plays a crucial role in machine learning, serving two fundamental purposes for end-users. Firstly, it serves as an effective and consistent method for communicating the quality of predictions to end-users, and secondly, as a measurable parameter facilitating the comparison and integration of diverse estimations of a target variable. Studies that use machine learning to generate predictions based on EO data do not routinely report uncertainty for several reasons, including a lack of consensus in methodologies, lack of accessible and easy-to-use tools, and a lack of access to computational power required for some methods^8,11^. We have demonstrated the utility of conformal prediction to quantify uncertainty in a robust and scalable manner relevant to EO-based applications that rely on classification and regression tasks.

A substantial proportion of the studies that quantified uncertainty were directed toward estimating sequestered carbon. This trend can be ascribed to the critical role of uncertainty quantification in carbon crediting. Various regulatory protocols (e.g., the Climate Action Reserve) have adopted a methodology wherein carbon credits are not allocated based on the median (point) estimate of sequestered carbon, but rather on the lower boundary of a prediction interval^39^. This approach is adopted to mitigate the risk of granting credits for carbon that has not genuinely been sequestered. While ensemble and quantile regression methods of quantifying uncertainty are generally accessible and straightforward to implement, it is important to note that neither of these approaches offer valid coverage guarantees under any data distribution and may result in a lower bound that is either over-conservative or overly optimistic.

Conformal prediction exhibits advantageous attributes for diverse machine learning applications, characterized by its model-agnostic nature, distribution-free characteristics, inherent validity, and computational efficiency, alongside straightforward implementation protocols. These attributes confer distinct advantages relative to alternative methodologies employed for UQ in EO (refer to Supplementary Table S1). Notably, conformal prediction enables the provision of valid quantitative uncertainty information, a capability surpassing that of quality flags that provide a qualitative assessment of the reliability of a prediction based on heuristics such as the number of pixels used to create a prediction. The Distance to Second Class (DS2C) method, employed for uncertainty quantification in random forest models, is exclusively applicable to decision-tree based models^40^. This heuristic notion of uncertainty, although limited to decision-tree based models, could concurrently serve as the foundation of a nonconformity score function that could be investigated in future work.

In contrast to UQ methodologies that requires the training of multiple models, such as those employed in quantile regression and ensemble strategies^41^, the split-conformal paradigm obviates the need for retraining the predictive model. Techniques like bootstrapping and ensemble methodologies are reliant upon differences in replicate model predictions to reflect high uncertainty^41^. Consequently, the training, evaluation, and inference stages incur significant computational costs, as multiple models need to be trained, and during evaluation and inference, inference must be repeated for each model^41^. Although parametric models like Gaussian processes (kriging) and Bayesian spatial models are commonly employed in spatial estimation and uncertainty quantification, they have not been utilized in any of the large-scale studies reviewed. This indicates difficulties in scaling to large and high-dimensional datasets. Furthermore, the provided uncertainty estimates lack distribution-free validity assurances and are not available in GEE.

The use of split-conformal prediction in scenarios characterized by extensive labelled datasets, representative of Dynamic World, will be more efficient if probability thresholds are made publicly available for various confidence levels. This will circumvent the calibration phase for new images or new users (refer to Supplementary Table S4). In contrast, when there is limited data, the statistical power of split conformal prediction will be reduced, producing conservative prediction regions with greater variance. Instead, the more sample efficient but more computationally demanding jackknife + or transductive conformal prediction methods should be used. However, increasing research efforts are likely to mitigate or minimise certain drawbacks associated with the conformal prediction framework. Furthermore, the continuous development of conformal prediction through active research, ensures its support for a diverse and expanding array of tasks, beyond regression^21^ and classification^11,23^, that include time series analysis^42^, semantic segmentation^43^ and diffusion models in generative AI^44^.

A limitation impeding the integration of conformal prediction into probabilistic machine learning for EO pertains to the lack of accommodation of post-processing procedures (for example, post classification filtering using erosion and dilation operations to reclassify isolated pixels or conditional radiance fields for semantic segmentation). Such post-processing procedures are often applied to the model's output without adjusting the underlying probability scores, resulting in a disparity between the improved model outputs and the unadjusted probability-derived non-conformity scores. Consequently, post-processing of predictions is likely to result in conservative prediction regions. To ensure statistical efficiency of the conformal predictor, it would therefore be advisable if post-processing steps could be performed as pre-processing steps when possible or dropped. Another drawback arises when the nonconformity scores derived for the calibration data lack exchangeability with those obtained during inference, potentially leading to prediction intervals with coverage below the user-defined confidence level (1-alpha). For example, this may occur under data drift scenarios resulting from changing atmospheric conditions, land use land cover change or climate change. This necessitates recalibration. Nevertheless, recent research has explored extensions to conformal prediction under distribution shifts, encompassing covariate and label shifts^45,46^.

Split conformal prediction provides an assurance of coverage on a population level, indicating marginal coverage across the entire dataset. This implies that certain subpopulations, delineated by categories or strata, may surpass the user-defined coverage, while others may fall short of the stipulated criterion^47^. Despite these drawbacks, conformal prediction is a compelling methodology with increasing potential to enhance the precision and dependability of machine learning systems. For instance, Mondrian conformal prediction or class-conditioned conformal prediction was introduced to extend the marginal coverage guarantee to encompass distinct classes or strata. This adjustment more closely aligns with the notion of conditional coverage, which mandates that each individual instance meets the prescribed coverage criteria^47^. Mondrian conformal prediction may be beneficial in cases whereby errors for specific classes are associated with higher real-world costs (for example, rare species or invasive species occurrences).

Operational machine learning systems are characterised by their capacity to consistently deliver datasets derived from satellites in an ongoing manner, thereby facilitating decision making. The successful integration of operational GeoAI systems and decision support systems depends on overcoming challenges that address the disparity between existing information provided by EO-derived products and the needs outlined by envisioned data users such as managers, policy makers, researchers, and field officers. Key among these challenges is the need for ongoing validation of a dataset post-release^48^. Data users frequently require insights into the accuracy and uncertainty of model predictions for a specific temporal and spatial context of relevance to them, irrespective of the spatio-temporal context of the validation data collected prior to dataset release. Conformal prediction allows uncertainty to be quantified for retrospective studies, models, and datasets at designated regions of interest that lack inherent uncertainty estimates, as illustrated for the Dynamic World dataset. This enables data users to assess the uncertainty of predictions for when and where it matters to them.

The inclusion of uncertainty information serves to mitigate a user’s over-reliance on less confident or low-quality predictions. This integration empowers users to identify instances, down to individual pixels, where the predictive model provides accurate information. Alternatively, as part of a quality control system, for low-quality predictions, users can defer to datasets of higher signal quality, engage a field officer, or consult local experts when faced with uncertainty. Similar approaches have been proposed for high-risk AI-health applications (for example,^49^). Here, the human-AI collaborative system learns the comparative precision of the predictive AI model in relation to a clinician's interpretation, and how that relationship fluctuates with the predictive AI’s confidence scores. Thereafter, for a new patient, the system evaluates the optimal course of action, weighing the AI’s decision against deferring to the clinician, with the overarching objective of determining the most accurate interpretation.

The identification of high uncertainty pixels may also expedite the transition towards collaborative human-AI systems, capitalizing on the synergistic benefits offered by each entity to overcome the limitations inherent in each individual system. The pixels that are flagged for human oversight may constitute the feedback that can contribute to an improvement cycle for the human-AI system, allowing continuous active learning^50^. For example, in the demonstrative case studies, there is increased uncertainty at object boundaries, such as water systems or forest patches (Fig. 4). Moreover, for canopy height estimation (Fig. 5), the uncertainty exhibits a notable increase for pixels containing image artifacts and for high reflectance pixels in the Terene desert. In this way, uncertainty represents a useful additional dimension when identifying a model’s failure cases. Understanding these shortcomings allows for system performance improvement through augmenting data collection with targeted field visits or labelling effort directed towards higher uncertainty multiclass sets, rectifying inaccuracies in labelling, optimizing feature selection, or by restricting the operational domain of the system^51^. For instance, knowing where uncertainty is greatest for invasive pine trees (pixels with multi-class sets that include the dense pine class, Fig. 4) helps allocate resources (time, budget or field equipment) more effectively. Resources can be concentrated on field visits that are likely to yield the most significant reductions in uncertainty. This will also directly improve the accuracy and reliability of model predictions, enhancing the overall quality of the model. Such a paradigm shift toward collaborative systems is anticipated to engender enhanced trust, increased adoption rates, and overall improved operational efficiency^49,50^.

In the context of the invasive species mapping case study, reference locality data were acquired from natural areas, with the deliberate exclusion of altered landscapes such as agricultural and urban environments. Consequently, increased uncertainty characterizes these anthropogenically altered regions. Conventional practice would entail restricting the operational scope by masking non-natural areas. Alternatively, the Area of Applicability method takes a more nuanced approach to restrict the scope of model inference. It relies on a dissimilarity index that measures the distance of a test pixel to each instance in the models’ training data, across all features^52^. However, this method has not yet gained traction in the EO community likely due to its limited availability in the R language, its lack of validity guarantees and high computational resource requirements. In contrast, the quantification of pixel-wise uncertainty provides users with the autonomy to assess if the increased uncertainty is acceptable. In the event of deemed unacceptability, users may explore alternative model or data-centric methodologies, previously mentioned, to reduce prediction uncertainty.

The validity property of conformal prediction could enhance the appeal of such systems for decision support frameworks relied upon by policymakers and land managers. In instances where the consequences of omission errors bear a higher cost than including erroneous data, as exemplified in wildfire monitoring and early detection of invasive species, a judicious approach involves the retrieval of all areas potentially associated with an introduction event, subject to a user-defined confidence level (95% probability). This would be a useful way to constrain omission errors despite the accuracy of the underlying model, thereby fostering the broader adoption of EO and data-driven decision-making practices.

The use of conformal prediction encourages the use of probabilistic machine learning. This may subsequently lead to positive outcomes for downstream data quality and for supporting a broader range of user needs. For example, numerous studies optimise classification systems using accuracy, precision, recall, F1-score, and more problematic metrics such as roc-auc and kappa that do not explicitly optimise prediction confidence and therefore model reliability^53,54^. In so doing, the user loses the opportunity to manually set the cost of omission and commission errors that are a function of model quality and a project’s goals. This potentially leads to misalignment between the provided model outputs and the user’s actual needs. Instead, binary cross-entropy (log loss) and categorical cross entropy should be preferred to avoid models that may be misaligned in comparison to their objectives. Both cross entropy losses penalize the incorrect classification based on prediction confidence, unlike metrics such as accuracy, precision, recall, and F1-score, which depend on discretised probability-like scores. For instance, in binary classification, a model may correctly predict a class with a probability score of 0.51, indicating high uncertainty that discretized metrics do not capture. For this reason, cross entropy loss is likely to generate more statistically efficient and informative prediction regions. Moreover, the provision of probability outputs (or at least uncalibrated pseudo-probabilities) should be encouraged^31^, as this will allow conformal prediction to be applied independently of the data provider, and irrespective of the access to the training data and inner workings of models. Google's Dynamic World dataset is a notable example of this^31^.

To facilitate the utilization of conformal prediction methodologies by researchers and practitioners in the EO domain, we present both Python and GEE JavaScript modules as a component of an extensive mapping pipeline, demonstrated for the invasive species mapping case study. The Python modules afford dual avenues for employing conformal prediction for new studies. First, users can use the native GEE implementations of the least ambiguous set-valued conformal classifier method for classification or the absolute residual method for regression tasks. This computational workflow was used for the dynamic world example. While we intend on supporting additional proven conformal methods natively for GEE, existing Python packages presently offer a more extensive array of approaches and may promptly accommodate new conformal prediction techniques. Consequently, we propose a second approach, wherein users can extract requisite data from GEE and seamlessly integrate it with pre-existing conformal prediction packages such as MAPIE^55^, which supports a range of conformal prediction methods. We illustrated the second workflow in the context of the canopy height regression task. This workflow is preferred for regression tasks since it allows for adaptive prediction intervals through quantile regression which is not yet natively supported in GEE. The code for all three case studies is provided as annotated Jupyter notebook-based tutorials to assist the adoption and adaptation of these methods in future studies. It is noteworthy that an extensive array of R, Python, and Julia packages exists to support conformal prediction; for a regularly updated compilation of resources, including textbooks, research papers, tutorials, and blogs related to conformal prediction refer to^56^.

### Future research directions

Contemporary trajectories in conformal prediction research revolve around the exploration of enhanced nonconformity scores that exhibit adaptability, statistical efficiency and valid coverage amidst distribution shifts^45^, label noise^57^, missing data^58^, nearing conditional coverage, all while maintaining computational efficiency and implementation simplicity (For example,^21,23^). The promise of conformal prediction and its associated validity and efficiency properties has spurred efforts among scholars to enhance established algorithms by combining them with conformal prediction. Noteworthy instances include the fusion with quantile regression^21^, explainable artificial intelligence, specifically Shapley Additive exPlanations (SHAP)^59^, and Monte Carlo prediction^60^. We hope to catalyse a parallel progression within the EO domain. Candidates for integration of conformal prediction include, change point detection, anomaly detection, active learning systems and continuous monitoring algorithms such as Continual Change Detection and Classification and Continuous Degradation Detection (CCDC and CODED, respectively)^61,62^.

The use of EO-derived datasets in data-driven decision-making has made a substantial contribution to the characterization, comprehension, and conservation of planet earth. Nevertheless, our examination of national to global scale datasets involved in these contributions highlights the lack of UQ accompanied by validity guarantees, and an absence of techniques capable of concurrently providing pixel-wise uncertainty information. We believe that UQ through the inclusion of conformal prediction into AI systems stands to significantly increase the role of EO data in operational monitoring systems, policy formulation, and regulatory reporting, accelerating progress towards the realisation of international planetary objectives and targets.

### Supplementary Information

Supplementary Information.

## Data Availability

The datasets used in the analysis are readily available in GEE as public assets and can be accessed and downloaded from GEE through the provided code (below).

## Code availability

The Python source code required for the reproduction of the three case studies are available at the following GitHub repository: https://github.com/Geethen/GEEConformal. This repository encompasses modules and illustrative examples facilitating the application of conformal prediction methodologies to novel studies. Additionally, the Google Earth Engine (GEE) JavaScript code pertinent to conformal prediction can be accessed via the following link: https://code.earthengine.google.com/?accept_repo=users/geethensingh/conformal.
