# Supplementary material for: Uncertainty quantification for probabilistic machine learning in earth observation using conformal prediction
Source: Sci Rep. 2024 Jul 13;14:16166. doi: 10.1038/s41598-024-65954-w (PMC11246475; doi:10.1038/s41598-024-65954-w)
Supplement: Supplementary file 1 — Supplementary Information. [file 41598_2024_65954_MOESM1_ESM.pdf]

Supplementary Information

Uncertainty quantification for probabilistic machine learning in earth observation using conformal prediction.

[1] Geethen Singh [*], [2, 3] Glenn Moncrieff, [4] Zander Venter, [5] Kerry Cawse-Nicholson, [6, 7] Jasper Slingsby and [1] Tamara B Robinson

[1] Centre for Invasion Biology, Department of Botany and Zoology, Stellenbosch University, South Africa

[2] Global Science, The Nature Conservancy, Cape Town, 7945, South Africa

[3] Centre for Statistics in Ecology, Environment and Conservation, Department of Statistical Sciences, University of Cape Town, Private Bag X3, Rondebosch 7701, South Africa

[4] Norwegian Institute for Nature Research—NINA, Sognsveien 68, 0855 Oslo, Norway

[5] Carbon Cycles and Ecosystems, Jet Propulsion Laboratory, California Institute of Technology, Pasadena, CA, United States

[6] Department of Biological Sciences and Centre for Statistics in Ecology, Environment and Conservation, University of Cape Town, Private Bag X3, Rondebosch 7701, South Africa

[7] Fynbos Node, South African Environmental Observation Network, Centre for Biodiversity Conservation, Cape Town, South Africa

Supplementary Table S1. An overview of the uncertainty methods used in the 17-machine learning-derived datasets made available through the Google Earth Engine (GEE) data catalogue and the GEE community catalogue. The datasets shown here represent 22.5% of the total machine learning-derived datasets examined.

Dataset	Uncertainty Quantification method	Spatio-temporal coverage	Reference
Soil carbon storage in terrestrial ecosystems of Canada	Quantile regression	National-Canada (N/A)	[1]
Irrecoverable carbon in Earth's ecosystems	Standard error of the uncertainty layers of the used datasets.	Global (2010, and 2018)	[2]
Soil Grids 250m v2.0	Quantile regression	Global (N/A)	[3]
Global Mangrove Project	Bootstrapping for confidence intervals around accuracy statistics	Global (1996, 2007-2010, 2015- 2020)	[4]
Land Change Monitoring, Assessment, and Projection (LCMAP) v1.3	Model Quality Flags includes persistent snow, insufficient data and clear conditions and Sample based area estimates.	National-CONUS (1985-2021)	[5]
ETH Global Sentinel-2 10m Canopy Height (2020)	Negative log likelihood loss function for aleatoric uncertainty and ensemble predictions for epistemic uncertainty.	Global (2020)	[6]
High Resolution Tree Species Information for Canada	Distance to second class (DS2C) based on 100*(1-nVotesC2/nVotesC1)	National-Canada (2019)	[7]
Canada Landsat Derived Forest harvest disturbance 1985-2020	DS2C based on 100*(1-nVotesC2/nVotesC1)	National-Canada (1985-2020)	[8]
Rangeland Analysis Platform layers (rangeland fractional cover)	Ensemble based prediction variance	National-CONUS (2019)	[9–11]
Ensemble Source Africa Cropland Mask 2016	Sample based area estimates	Continental-Africa (2016)	[12]
Highly Scalable Temporal Adaptive Reflectance Fusion Model (HISTARFM) database	Kalman filter	National-CONUS (2009-2021)	[13]
Global Photovoltaics Inventory (2016-2018)	Custom mechanistic approach which makes distribution assumptions and bootstrapping.	Global (2016-2018)	[14]
Canada Landsat Derived Wildfire disturbance & Magnitude 1985-2020	(DS2C) based on 100*(1-nVotesC2/nVotesC1)	National-Canada (1985-2020)	[8]
RADD Forest Disturbance Alert	Probabilistic mapping using Gaussian mixture models and Bayesian methods	Global (2019-2020)	[15]
iSDASoil	Quantile regression and bootstrapping.	Continental – Africa (2021)	[16]

SoilGrids v2.0.	Quantile regression	Global (N/A)	[3]
Global urban projections under SSPs (2020-2100)	Ensemble based prediction variance	Global (2020-2100)	[17,18]
Murray Global Intertidal Change	Quality flags that contain the number of input pixels for modelling.	Global (1984-2016 in 3-year intervals)	[19]
USDA NASS cropland data layers	A hybrid ensemble expert voting system.	National-CONUS (1997-2023)	[20]
MCD12Q1.061 MODIS Land Cover Type Yearly Global 500m	A hybrid ensemble expert voting system.	Global (2001-2022)	[21]

Supplementary Table S2. A list of datasets from the Google Earth Engine (GEE) community catalogue that are not derived using machine learning and are therefore not assessed for uncertainty quantification. This list considered datasets made available up to the 2 November 2023 update.

No.	Dataset name
1	GPW Version 4 adminstration units
2	geoBoundaries Global Database of Political Administrative Boundaries
3	Edge-matched Global, Subnational and operational Boundaries
4	West Africa Coastal Vulnerability Mapping
5	Social Connectedness Index (SCI)
6	Gridded Global GDP and HDI (1990-2015)
7	Harmonized Global Critical infrastructure & Index (CISI)
8	Native Land (Indigenous Land Maps)
9	Gridded Sex-Disaggregated School-Age Population (2020)
10	USA Structures
11	Geomorpho90m Geomorphometric Layers
12	Bare Earth's Surface Spectra 1980-2019
13	Normalized Sentinel-1 Global Backscatter Model Land Surface
14	Soil nematode abundance & functional group composition
15	Global maps of habitat types
16	Global Surface water and groundwater salinity measurements (1980-2019)
17	Copernicus Digital Elevation Model (GLO-30 DEM)
18	ASTER Global Digital Elevation Model (GDEM) v3
19	ASTER Global Water Bodies Database (ASTWBD) Version 1
20	General Bathymetric Chart of the Oceans (GEBCO)
21	Coastal National Elevation Database (CoNED) Project -Topobathymetric digital elevation models (TBDEMs)
22	NOAA Sea-Level Rise Digital Elevation Models (DEMs)

23	ÍslandsDEM v1.0 10m
24	DEM France (Continental) 5m IGN RGE Alti
25	Soil Properties 800m
26	Polaris 30m Probabilistic Soil Properties US
27	HiHydroSoil v2.0 layers
28	Global Soil bioclimatic variables
29	Harmonized World Soil Database (HWSD) version 2.0
30	Global Mangrove Distribution, Aboveground Biomass, and Canopy Height
31	ESA WorldCover 10 m 2020 V100 InputQuality
32	LandCoverNet Training Labels v1.0
33	CloudSEN12 - Global dataset for semantic understanding of cloud and cloud shadow in Sentinel-2
34	West Africa Land Use Land Cover
35	Mississippi River Basin Floodplain Land Use Change (1941-2000)
36	OSM Water Layer Surface Waters in OpenStreetMap
37	Global 30m Height Above the Nearest Drainage
38	Hydrography 90m Layers
39	HydroLAKES v1.0
40	HydroATLAS v1.0
41	HydroWaste v1.0
42	Global River Width from Landsat (GRWL)
43	DynQual Global Surface Water Quality Dataset
44	Global coastal rivers and environmental variables
45	Global River Deltas and vulnerability
46	Streamflow reconstruction for Indian sub-continental river basins 1951–2021
47	Global georeferenced Database of Dams (GOODD)
48	RealSAT Global Dataset of Reservoir and Lake Surface Area
49	Global Hydrologic Curve Number (GCN250)
50	Global high-resolution floodplains (GFPLAIN250m)
51	Global river networks & Corresponding Water resources zones
52	National Wetland Inventory (Surface Water and Wetlands)
53	National Hydrography Dataset (NHD)
54	Digital Earth Australia Coastlines
55	Digital Earth Africa Coastlines
56	Argo Float Data (Subset)
57	Global gridded sea surface temperature (SSTG)
58	Global Storm Surge Reconstruction (GSSR) database
59	Aqualink ocean surface and subsurface temperature subset
60	Plastic Inputs from Rivers into Oceans
61	Mismanaged Plastic Waste Dataset in Rivers

62	Global Ocean Data Analysis Project (GLODAP) v2.2022
63	USGS VIIRS Evapotranspiration
64	USGS MODIS Evapotranspiration
65	NOAA Evaporative Stress Index (ESI)
66	Forecast Reference Crop Evapotranspiration (FRET)
67	Global Forest Carbon Fluxes (2001-2022)
68	USDA Crop Sequence Boundaries 2015-2022
69	ESA CCI Global Forest Above Ground Biomass
70	geeSEBAL-MODIS Continental scale ET for South America
71	Global Fungi Database
72	Global Long-term Microwave Vegetation Optical Depth Climate Archive (VODCA)
73	Global Sunlit and Shaded GPP for vegetation canopies (1992-2020)
74	Aboveground carbon accumulation in global monoculture plantation forests
75	Benchmark maps Secondary Forest Brazil
76	NAFD Forest Disturbance History 1986-2010
77	Tile Drained Croplands (30m)
78	Global crop production tillage practices
79	Global Fertilizer usage by crop & country
80	Open Aerial Map Subset
81	HySpecNet-11K Hyperspectral Benchmark dataset
82	Santa Rita Experimental Range Drone Imagery
83	USGS Historical Topo Maps
84	USGS Historical Imagery Western US
85	Global Power
86	Facebook Electrical Distribution Grid Maps
87	Harmonized Global Night Time Lights (1992-2021)
88	Global Roads Inventory Project
89	Global Highres Mining Footprints
90	Global Mining Areas and Validation Datasets
91	Global Healthsites Mapping Project
92	Global fixed broadband and mobile (cellular) network performance
93	Global Power Plant Database
94	Global offshore wind turbine dataset
95	Harmonised global datasets of wind and solar farm locations and power
96	Global Database of Cement Production Assets
97	Global Consensus Landcover
98	Global Freshwater Variables
99	Global Habitat Heterogeneity
100	Global 1-km Cloud Cover

101	Areas of global conservation value
102	CEMS Fire Danger Indices
103	Wildfire Risk to Communities (WRC)
104	Global Fire WEather Database (GFWED)
105	Global Fire Atlas (2003-2016)
106	Archival NRT FIRMS Global VIIRS and MODIS vector data
107	Monitoring Trends in Burn Severity (MTBS) 1984-2019
108	Global large flood events (1985-2016)
109	Global Landslide Catalog (1970-2019)
110	MAXAR Open Data Events
111	Umbra SAR Open Data
112	Geocoded Disasters (GDIS) Dataset (1960−2018)
113	Global Reference Evapotranspiration Layers
114	Global Aridity Index
115	Global Wind Atlas Datasets
116	Global Solar Atlas Datasets
117	Global Extreme Heat Hazard
118	New improved Brazilian daily weather gridded data (1961–2020)
119	International Satellite Cloud Climatology Project HXG Cloud Cover
120	Current and projected climate data for North America (CMIP6 scenarios)
121	Terraclimate Individual years for +2C and +4C climate futures
122	Global MODIS-based snow cover monthly values (2000-2020)
123	MOD10A2061 Snow Cover 8-Day L3 Global 500m
124	MODIS Gap filled Long-term Land Surface Temperature Daily (2003-2020)
125	Global Daily near-surface air temperature (2003-2020)
126	Snow Data Assimilation System (SNODAS)
127	United States Drought Monitor Layers
128	North American Drought Monitor (NADM)
129	Canadian Drought Outlook
130	United States Seasonal Drought Outlook
141	Global Precipitation Measurement (GPM)
142	ANUSPLIN Gridded Climate Dataset
143	High Resolution Deterministic Prediction System (HRDPS)
144	Regional Deterministic Precipitation Analysis (RDPA)
145	Regional Deterministic Prediction System (RDPS)
146	Climate Prediction Center (CPC) Morphing Technique (MORPH)
147	Modern-Era Retrospective analysis for Research and Applications, Version 2 (MERRA2)
148	Applied Climate Information System (ACIS) NRCC NN
149	Climate Hazards Group InfraRed Precipitation with Station Data-Prelim (CHIRPS-Prelim)

150	NOAA Monthly U.S. Climate Gridded Dataset (NClimGrid)
151	High-spatial-resolution Thermal-stress Indices over South and East Asia (HiTiSAE)
152	Reference ET gridded database based on FAO Penman-Monteith for Peru (PISCOeo_pm)
153	Daylight Map Distribution map data
154	Global human modification v1.5

Supplementary Table S3. A list of datasets from the Google Earth Engine (GEE) community catalogue and main catalogue (*) that are derived using machine learning but do not quantify uncertainty and are therefore not assessed for their uncertainty quantification method (Table 1). This list considered datasets made available up to the 2 November 2023 update.

No.	Dataset name
1	High resolution settlement layer
2	Landscan
3	Relative Wealth Index (RWI)
4	Global Human Settlement Layer 2023
5	Global ML Building Footprints
6	Global Electric Consumption revised GDP
7	Soil Organic Carbon Stocks & Trends South Africa
8	FABDEM (Forest And Buildings removed Copernicus 30m DEM)
9	Global Soil Salinity Maps (1986-2016)
10	ESRI 10m Annual Land Use Land Cover (2017-2022)
11	GlobCover Global Land Cover
12	Finer Resolution Observation and Monitoring of Global Land Cover 10m (FROM-GLC10)
13	Global Impervious Surface Area (1972-2019)
14	Global urban extents from 1870 to 2100
15	World Settlement Footprint & Evolution
16	Mapbiomas Annual land cover and use maps
17	CCI LAND COVER S2 PROTOTYPE LAND COVER 20M MAP OF AFRICA 2016
18	South African National Land Cover (SANLC)
19	Digital Earth Australia (DEA) Landsat Land Cover 25m v1.0.0
20	UrbanWatch 1m Land Cover & Land Use
21	Vermont High Resolution Land Cover 2016
22	Chesapeake Bay High Resolution Land Cover Dataset (2013-2014)
23	C-CAP High-Resolution Land Cover

24	C-CAP Medium-Resolution Land Cover - Beta
25	C-CAP Wetland Potential 30m
26	Oil Palm Plantation Layers
27	Rasterized building footprint dataset for the US
28	Global River Classification (GloRiC)
29	GLOBathy (Global lakes bathymetry dataset)
30	High-Res water body dataset for tundra and boreal forests North America
31	Global Channel Belt (GCB)
32	Tensor Flow Hydra Flood Models
33	High-resolution gridded precipitation dataset for Peruvian and Ecuadorian watersheds (1981-2015)
34	Global Shoreline Dataset
35	Landfire Mosaics LF v2.2.0
36	Vegetation dryness for western USA
37	GIMMS Normalized Difference Vegetation Index 1982-2022
38	High-resolution annual forest land cover maps for Canada's forested ecosystems (1984-2019)
39	Canopy height forested ecosystems of Canada
40	Canada Landsat derived FAO forest identification (2019)
41	Landsat-derived forest age for Canada's forested ecosystems
42	US National Forest Type and Groups
43	Global Forest Canopy Height from GEDI & Landsat
44	Global Forest Management dataset 2015
45	Global 30m Landsat Tree Canopy Cover v4
46	Global tree allometry and crown architecture (Tallo) database
47	Global Leaf trait estimates for land modelling
48	NASA Harvest Layers
49	Digital Earth Africa's cropland extent map Africa 2019
50	GFSAD Global Cropland Extent Product (GCEP)
51	GFSAD Landsat-Derived Global Rainfed and Irrigated-Cropland Product (LGRIP)
52	Global irrigation areas (2001 to 2015)
53	Global NPP-VIIRS-like nighttime light (2000-2022)
54	Global database of cement production assets and upstream suppliers
55	Global Database of Iron and Steel Production Assets
56	Biodiversity Intactness Index (BII)
57	30m Global Annual Burned Area Maps (GABAM)
58	AgERA5 (ECMWF) dataset
59	Long-term Gap-free High-resolution Air Pollutants (LGHAP)
60	POMELO Model Population Density Maps
61	Global Intra-Urban Land Use

62	Continental-scale land cover mapping at 10 m resolution over Europe
63	*Allen coral atlas
64	*WorldPop Global Project Population Data
65	*GEOS-CF fcst tavg1hr v1
66	*IrrMapper
67	*Dynamic world
68	*Open LandMap layers
69	*Global Human Settlement layers (GHSL)

Supplementary Table S4. Pre-computed qHat thresholds at various confidence levels (1-α) for Google Dynamic World based on a random 50% calibration set.

Confidence level	qHat
0.95	0.03718
0.90	0.06068
0.85	0.08787
0.80	0.11914
0.75	0.16599
0.70	0.22468

References

1. Sothe, C. *et al.* Large soil carbon storage in terrestrial ecosystems of Canada. *Global Biogeochem Cycles* **36**, e2021GB007213 (2022).

2. Noon, M. L. *et al.* Mapping the irrecoverable carbon in Earth's ecosystems. *Nat Sustain* **5**, 37–46 (2022).

3. Poggio, L. *et al.* SoilGrids 2.0: producing soil information for the globe with quantified spatial uncertainty. *Soil* **7**, 217–240 (2021).

4. Bunting, P. *et al.* Global mangrove extent change 1996–2020: Global mangrove watch version 3.0. *Remote Sens (Basel)* **14**, 3657 (2022).

5. Brown, J. F. *et al.* Lessons learned implementing an operational continuous United States national land change monitoring capability: The Land Change Monitoring, Assessment, and Projection (LCMAP) approach. *Remote Sens Environ* **238**, 111356 (2020).

6. Lang, N., Jetz, W., Schindler, K. & Wegner, J. D. A high-resolution canopy height model of the Earth. *Nat Ecol Evol* 1–12 (2023).

7. Hermosilla, T., Bastyr, A., Coops, N. C., White, J. C. & Wulder, M. A. Mapping the presence and distribution of tree species in Canada's forested ecosystems. *Remote Sens Environ* **282**, 113276 (2022).

8. Hermosilla, T. *et al.* Mass data processing of time series Landsat imagery: pixels to data products for forest monitoring. *Int J Digit Earth* **9**, 1035–1054 (2016).

9. Robinson, N. P. *et al.* Rangeland productivity partitioned to sub-pixel plant functional types. *Remote Sens (Basel)* **11**, 1427 (2019).

10. Jones, M. O. *et al.* Annual and 16-day rangeland production estimates for the western United States. *Rangel Ecol Manag* **77**, 112–117 (2021).

11. Allred, B. W. *et al.* Improving Landsat predictions of rangeland fractional cover with multitask learning and uncertainty. *Methods Ecol Evol* **12**, 841–849 (2021).

12. Nabil, M., Zhang, M., Wu, B., Bofana, J. & Elnashar, A. Constructing a 30m African Cropland Layer for 2016 by Integrating Multiple Remote sensing, crowdsourced, and Auxiliary Datasets. *Big Earth Data* **6**, 54–76 (2022).

13. Moreno-Martínez, Á. *et al.* Multispectral high resolution sensor fusion for smoothing and gap-filling in the cloud. *Remote Sens Environ* **247**, 111901 (2020).

14. Kruitwagen, L. *et al.* A global inventory of photovoltaic solar energy generating units. *Nature* **598**, 604–610 (2021).

15. Reiche, J. *et al.* Forest disturbance alerts for the Congo Basin using Sentinel-1. *Environmental Research Letters* **16**, 24005 (2021).

16. Hengl, T. *et al.* African soil properties and nutrients mapped at 30 m spatial resolution using two-scale ensemble machine learning. *Sci Rep* **11**, 6130 (2021).

17. Chen, G. *et al.* Global projections of future urban land expansion under shared socioeconomic pathways. *Nat Commun* **11**, 537 (2020).

18. Gao, J. & O'Neill, B. C. Mapping global urban land for the 21st century with data-driven simulations and Shared Socioeconomic Pathways. *Nat Commun* **11**, 2302 (2020).

19. Murray, N. J. *et al.* The global distribution and trajectory of tidal flats. *Nature* **565**, 222–225 (2019).

20. NASS, U. USDA national agricultural statistics service cropland data layer. *USDA-NASS, Washington, DC* (2016).

21. Friedl, M. & Sulla-Menashe, D. MODIS/Terra+ Aqua land cover type yearly L3 global 500m SIN grid V061. *NASA EOSDIS Land Processes DAAC* (2022).
